# Potential of short‐term heat‐treated horse manure as a recycled growing media

**DOI:** 10.1002/jeq2.70099

**Published:** 2025-10-14

**Authors:** Salla Leppäkoski, Vilhelmiina Harju, Marika Tossavainen, Ilpo Pölönen

**Affiliations:** ^1^ HAMK Bio Research Unit Häme University of Applied Sciences Hämeenlinna Finland

## Abstract

This study evaluated the effectiveness of a farm‐scale manure pasteurizer in transforming horse manure into growing media for plants within a circular economy framework. Horse manures with wood or peat beddings were homogenized in a controlled short‐term heat treatment process lasting a total of 1–2 days, including a 1 h pasteurization phase. The elimination of harmful microorganisms and seed viability were studied prior to and after sanitation. The phytotoxicity and fertilization effect of the treated manures were evaluated. This study indicated a substantial reduction in *Escherichia coli*, from over 5.2 × 10^4^ colony‐forming unit (CFU) g^−1^ in untreated manure to below 173 CFU g^−1^​ in pasteurized manure, and complete loss of germination in four cereal seeds and a weed seed mixture tested. In a phytotoxicity test conducted using cress (*Lepidium sativum* L.), 20% of pasteurized horse manure with bedding was well tolerated, whereas phytotoxic symptoms were detected in treatments containing 50% of untreated horse manure or pasteurized horse manure (PHM) with wood or peat bedding and in the treatment with 20% PHM with wood bedding. In the growing experiment with Chinese cabbage [*Brassica rapa* L. subsp*. pekinensis* (Lour.) Hanelt], a fertilization effect was indicated despite the low rate of nitrogen efficiency. The promising application of the short‐term heat treatment process highlights its potential to improve sustainable farming, in line with the principles of the circular economy, by recycling horse manure into a growing media.

## Core Ideas

- Horse manure can be reused as a growing media after pasteurization.
- Pasteurization effectively eliminates microbes and reduces the viability of seeds existing in horse manure.
- Phytotoxic symptoms may occur with high concentrations of pasteurized horse manure.
- The nitrogen in pasteurized horse manure with peat bedding is not readily bioavailable in short‐term cultivation.

## Plain Language Summary

Nowadays, horse stables are often located near cities, making manure circulation to fields challenging. We evaluated pasteurization as a method for manure treatment and the suitability of sanitized manure mixed with peat for plant cultivation. Pasteurization effectively sanitizes manure, making it suitable for plant cultivation at low concentrations. Pasteurized horse manure can partially replace peat in growing media, promoting sustainability goals in agriculture and conserving natural resources.

**Abbreviations**
AGR: average germination rate
ARLP: average root length per plant
CFU: colony‐forming unit
MLV: Munoo–Liisa vitality index
PFPN: partial factor productivity of nitrogen
PHM: pasteurized horse manure
UHM: untreated horse manure

## 1 INTRODUCTION

There are approximately six million horses in European Union (EU) countries (EHN, 2024). On average, each horse produces 17 kg of manure daily (Westendorf & Krogmann, 2006). Due to urbanization, horse stables are nowadays located closer to cities, which increases the need to address the environmental challenges related to horse management, that is, nutrient recycling and waste management (Rantala et al., 2018). Manure handling is problematic, especially for stables without any farmland. Horse manure is classified as biowaste in the EU and should therefore be utilized primarily as fertilizer or, secondarily, as an energy source (Airaksinen, 2006).

Horse manure collected from stables is a mixture of manure, urine, bedding material, and a small amount of hay. Its composition varies depending on the stable, as different types of bedding are used. Common bedding materials include peat, peat moss, wood shavings, sawdust, wood pellets, and straw (Keskinen et al., 2017; Kwiatkowska‐Stenzel et al., 2016). Bedding material influences the welfare of horses and should therefore be of high quality to ensure adequate hygienic and microclimatic conditions in the stable (Kwiatkowska‐Stenzel et al., 2016). The absorption and retention of ammonia and other nutrients, for example, phosphorus and potassium, is essential when used as bedding, but nutrients should also be released when manure is recycled into agricultural soil as fertilizer or soil amendment (Keskinen et al., 2017).

Due to the shortage of bedding materials and issues with manure disposal, the possibility of reusing part of the manure as horse bedding or as growing media has been considered, homogenizing and pasteurizing it. To achieve this, the manure must undergo a heating process, known as pasteurization, which sanitizes it at temperatures below 100°C for a period of time that depends on the temperature used (Regulation (EU) 2019/1009).

Nowadays, composting is the most common method for manure sanitation. It has many benefits, including reducing the volume, moisture content, and odor of manure, increasing nutrient concentrations, and eliminating nitrogen immobilization (Keskinen et al., 2017). Composting is a slow process, and bedding material is not always composted adequately or efficiently (Airaksinen et al., 2001). It requires composting facilities that stables located near urban centers do not have. However, once horse manure has been sanitized, it can be partially reused as bedding material or sold from the stable, for example, for use as a soil amendment or even as a growing media.

It has been estimated that global demand for growing media for horticultural purposes will increase over the following 25 years. At present, it stands at 67 Mm^3^, of which peat accounts for 40 Mm^3^ (Blok et al., 2021). Concomitantly, due to climate change, the use of peat should be reduced and replaced by other materials in the future. One way to tackle environmental issues is to reuse and recycle materials for different purposes. For example, peat‐based growing media have been successfully reused in greenhouse cultivation after a steam sanitation process (van Loenen et al., 2003; Vandecasteele et al., 2020, 2024).

Since horse manure contains both manure and bedding material, it can be considered both a growing media and a nutrient reserve for plants. The bedding material used in stables influences the characteristics and nutrient content of horse manure. Nitrogen mineralization has been found to be low in horse manure with peat, straw, and wood bedding (Clark & Cavigelli, 2005; Keskinen et al., 2017), which can limit plant growth. However, composted horse manure, including peat bedding, has been successfully used as a growing media for vegetables in greenhouse without any loss of yield or contamination by fecal microbes (Airaksinen, 2006).

The challenges posed by composting of horse manure in urban areas due to inappropriate composting or storage facilities or insufficient reduction of human pathogens (Heinonen‐Tanski et al., 2006; Romano et al., 2006) create a need to explore alternative sanitation methods that are both energy efficient and fast. In colder climate conditions, the composting process may even be halted during winter months due to reduced microbial activity (Keskinen et al., 2017). In addition to composting, other methods of horse manure sanitation include thermal drying, pyrolysis, incineration, and anaerobic digestion (Eriksson et al., 2016; Luostarinen et al., 2020). The disadvantages of these methods are high electricity consumption and high capital, labor, or operating costs (Heinonen‐Tanski et al., 2006). Pasteurization has previously been used to sanitize slurry or solid manure for biogas production (Heinonen‐Tanski et al., 2006; Michailidou et al., 2024), but to our knowledge, not for the production of growing media.

This study evaluated the suitability of short‐term heat treatment for the sanitation of horse manure and the use of pasteurized horse manure as a growing media. Bacterial growth, seed viability, and phytotoxicity of horse manure with two different bedding materials, wood and peat, were determined in untreated horse manure (UHM) and pasteurized horse manure (PHM). In addition, a greenhouse growing experiment was conducted using PHM with peat bedding as a partial growing media to determine the fertilizing effect of nitrogen existing in PHM.

## 2 MATERIALS AND METHODS

### 2.1 Technology of manure pasteurization

**FIGURE 1:**
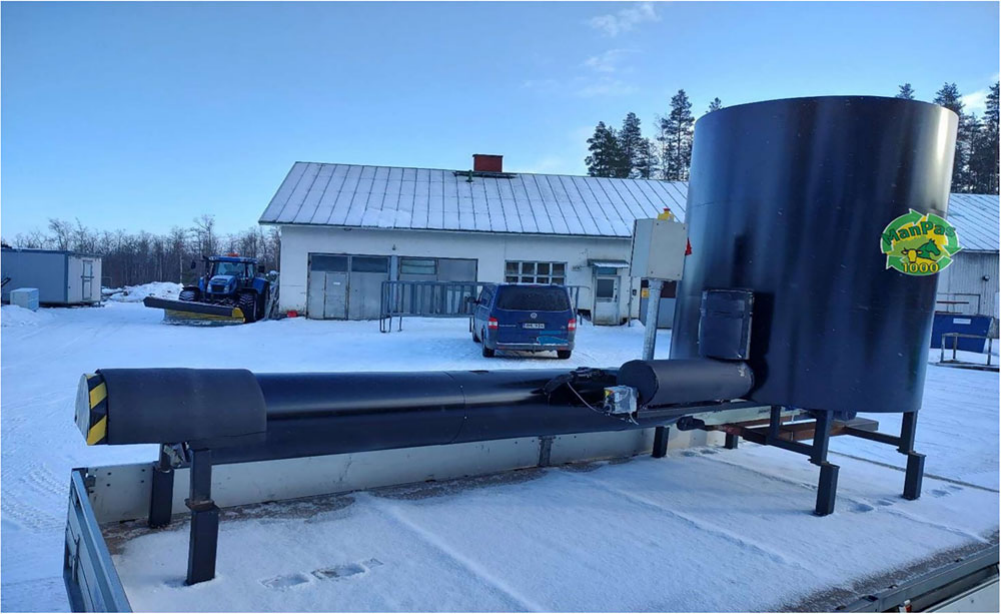
Prototype of the ManPas 1000 manure pasteurization device for horse manure and cow slurry solids. Photo: Ilpo Pölönen.

The ManPas manure pasteurization device (Figure 1) used in this study utilizes the heat generated by microbial decomposition of manure to heat it to 70°C for at least 1 h. This meets the hygiene standards set by the EU for manure (Regulation (EU) 2019/1009). The manure passes through the device in 1 or 2 days, and the pasteurization process itself lasts just 1 h.

The device consists of a silo and a discharge screw conveyor. In this automated operating system, the manure is moved by the screw once the desired temperature is reached and maintained. Temperature sensors (TEAT PT 1000; Produal Oy) monitor the pasteurization phase. After pasteurization, the product is bedding‐like and homogenous, with no visible fecal balls.

**Core Ideas**

- Horse manure can be reused as a growing media after pasteurization.
- Pasteurization effectively eliminates microbes and reduces the viability of seeds existing in horse manure.
- Phytotoxic symptoms may occur with high concentrations of pasteurized horse manure.
- The nitrogen in pasteurized horse manure with peat bedding is not readily bioavailable in short‐term cultivation.

### 2.2 Experimental setup

**FIGURE 2:**
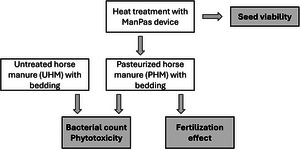
Experimental setup for evaluating the suitability of pasteurized horse manure as a growing media.

The study was conducted through a series of experiments, which are shown in Figure 2. Bacterial (*Escherichia coli*) count and phytotoxicity were analyzed from UHM and PHM with wood or peat bedding. The effect of pasteurization on seed viability was evaluated using cereal seeds and a weed seed mixture. The growing experiment in greenhouse conditions to determine fertilization effect was carried out with PHM with peat bedding.

### 2.3 Source and composition of the horse manure

Horse manures were collected from two horse stables in the Forssa region of southern Finland. One stable used wood bedding, and the other used peat bedding. The manure with bedding materials was <1‐week old when collected for the experiments. Half of the collected materials were pasteurized, and the other half were left untreated. Horse manures were collected in April and December 2022. The batch collected in April 2022 was used for the *E*. *coli* counts and phytotoxicity tests, and the batch collected in December 2022 was used for the growing experiment. The storing temperature after collection and pasteurization of UHM and PHM was 5°C. The dry matter content (DM, %), bulk density (Mg m^−3^), and total amount of nitrogen (N‐tot, g kg^−1^ DM) of UHM and PHM were analyzed before the experiments by Eurofins Agro (Finland) and are presented in Table 1.

**TABLE 1:** Dry matter content (DM, %), bulk density (Mg m^−3^), and total amount of nitrogen (N‐tot, g kg^−1^ DM) of untreated or pasteurized horse manure (UHM or PHM, respectively) with wood or peat bedding used in the phytotoxicity test and growing experiment.

Treatment	DM (%)	Bulk density (Mg m^−3^)	N‐tot (g kg^−1^ DM)
Phytotoxicity test			
UHM with wood bedding	26.2	0.28	13
PHM with wood bedding	39.4	0.23	21
UHM with peat bedding	24.6	0.32	19
PHM with peat bedding	33.2	0.23	20
Growing experiment			
PHM with peat bedding	37.2	0.42	18

### 2.4 Bacterial count (*E. coli*)

Six batches of PHM with peat bedding and four batches of PHM with wood bedding were analyzed for *E. coli*. Six batches of untreated (UHM) samples were collected from peat bedding only.

For the *E. coli* analysis, samples of 5 g were first homogenized with 45 mL of sterile 0.1% peptone salt solution in homogenizer blender bags using MiniMix Homogenizer (Interscience, France). The presence of *E*. *coli* was analyzed using 3 M Petrifilm *E. coli*/Coliform Count Plates (3 M). Dilution series of the homogenized samples in peptone solution was prepared at concentrations ranging from 10^−1^ to 10^−7^ for UHM and from 10^−1^ to 10^−4^ for PHM. For each dilution, a 1 mL sample was pipetted onto the count plate, and the plates were incubated for 24 h at 35°C, after which the blue colonies with gas bubbles were counted as *E. coli*. The plates were re‐incubated for an additional 24 h at 35°C, and the *E. coli* colonies were counted again. The detection limit for *E*. *coli* counts was set to 10 CFU g^−1^ (where CFU is colony‐forming unit). All samples were analyzed in triplicate.

### 2.5 Influence of pasteurization on seed viability

A germination test was used to evaluate the suitability of pasteurization for the removal of viable seeds commonly found in horse manure. One hundred seeds of barley (*Hordeum vulgare* L. subsp. *vulgare*), oat (*Avena sativa* L.), rye (*Secale cereale* L.), or wheat (*Triticum aestivum* L. subsp. *aestivum*), or 1 g of a weed seed mixture containing cereal seeds and weed seeds (a byproduct of a cereal dryer) were sealed in nylon bags with a pore size of 40 µm. The nylon bags were applied to the ManPas device filled with horse manure. The experiments were carried out with materials treated at >70°C for at least 60 min. Meanwhile, control treatment seeds were stored at room temperature (22°C) without pasteurization before the germination test.

The temperature during the pasteurization process was measured using dataloggers (Track‐IT Rugged Temp Data Logger; Monarch Instrument) placed in the nylon bags containing the seeds. After the pasteurization process, the nylon bags were collected from the horse manure for germination testing. All treatments were carried out with three replicates.

For the germination test, the seeds were taken out of the nylon bags and spread out on filter paper, covered with another piece of filter paper, and rolled up in a scroll. The scrolls were put in a plastic bag with a small ventilation hole on the side. The seeds were allowed to germinate for 4 days in the refrigerator (5°C) and then at room temperature (22°C) for 3–5 days. For barley, oat, rye, and wheat, the number of germinated seeds was counted when the sprouts were 6–8 cm in length. The number of germinated and non‐germinated seeds of weed seed mixture was counted in three 5 × 5 cm squares. The percentage of germinated seeds was considered to be the germination rate (%) of the sample. One of the replicated samples in the wheat germination test was excluded from the germination rate count, since it did not reach the required 1‐h exposure time at 70°C during the pasteurization.

### 2.6 Determination of phytotoxicity

The phytotoxicity of UHM and PHM with wood or peat bedding was evaluated using the standard method SFS‐EN 16086‐2:2011 (2012). This phytotoxicity test is based on the determination of cress (*Lepidium sativum* L.) seed germination and root development inhibition in comparison to control treatment.

The experiments were carried out on UHM and PHM with two different bedding materials, wood and peat, which were mixed with horticultural peat growing media (Kekkilä VHM 620, pH 6.0 R8060; Kekkilä‐BVB) in two different volumes (20% and 50%, v/v). The same peat (100%) was used as a control treatment. All treatments were carried out with five replicates. Prior to the experiments, the prepared samples were moistened with tap water to optimal moisture content for growth, following the method described in the standard.

The phytotoxicity tests were carried out in Petri dishes (100 × 100 × 15 mm; Phoenix Biomedical Products) with 150 mL of the mixture. Ten cress seeds (Hyötykasviyhdistys ry) were sown in each Petri dish, and the Petri dishes were incubated in darkness in a growing chamber (FitoClima 600PLH; Aralab) at 25°C for 72 h. The number of germinated seeds was counted, and the root lengths were measured after incubation. The average germination rates (AGRs, %) were calculated using the germination rates of each replicate. The average root length per plant (ARLP, mm) were calculated using Equation (1).

(1) $$\mathrm{ARLP}\left(\mathrm{mm}\right)=\frac{\frac{\sum RL_{1}}{\mathrm{NGS}}+\frac{\sum RL_{2}}{\mathrm{NGS}}+\cdots +\frac{\sum RL_{5}}{\mathrm{NGS}}}{5}$$

where *RL*_1_, *RL*_2_, *RL*_3_, *RL*_4_, and *RL*_5_ are the root lengths per plant in each replicate, and NGS is the number of germinated seeds in each replicate.

The Munoo–Liisa vitality index (MLV) was calculated using Equation (2). The MLV accounts for both the germination rate and the root lengths of germinated seeds compared to the control treatment.

(2) $$\begin{array}{ccc} & & MLV\left(\%\right)=\\ & & \frac{\left(GR_{1}\cdot RL_{1}\right)+\left(GR_{2}\cdot RL_{2}\right)+\cdots +\left(GR_{5}\cdot RL_{5}\right)}{5\cdot \left(GR_{C}\cdot RL_{C}\right)}\\ & & \cdot 100 \end{array}$$

where *GR*_1_, *GR*_2_, *GR*_3_, *GR*_4_, and *GR*_5_ are the germination rates in each replicate, *GR*_c_ is the AGR in the control treatment, *RL*_1_, *RL*_2_, *RL*_3_, *RL*_4_, and *RL*_5_ are the root lengths per plant in each replicate, and *RL*_c_ is the average root length in the control treatment.

### 2.7 Determination of fertilization effect

In the growing experiment, the fertilization effect of PHM with peat bedding was tested using OECD Guidance Document No. 227 for determining chemical properties (OECD, 2006). Chinese cabbage [*Brassica rapa* L. subsp. *pekinensis* (Lour.) Hanelt ‘Sprinkin’ F1] (Berner Oy) was used as a test crop. The duration of the growing experiment was 32 days. The experiment was terminated before the Chinese cabbages began forming heads.

The growing experiment was carried out in a greenhouse with six treatments, including the control treatment. The treatments were prepared by mixing PHM with peat (Kekkilä VHM 420, pH ∼ 6.0) in volumes of 0% (control treatment), 10%, 20%, 30%, or 40% v/v (hereafter referred to as 10% PHM, 20% PHM, 30% PHM, and 40% PHM treatments). Additional nitrogen fertilizer (CaNO_3_, NPK 15.5‐0‐0; Van Iperen Calcium Nitrate Horticultural Grade) was added in one treatment (hereafter referred to as 10% PHM + added N treatment) to equalize the total nitrogen level with the control treatment. The total amount of nitrogen in the treatments containing manure (10% PHM, 10% PHM + added N, 20% PHM, 30% PHM, and 40% PHM) was 0.281–1.125 kg m^−3^ (Table 2). For the control treatment, 2 kg m^−3^ of controlled‐release fertilizer (Osmocote Exact 3–4 M, NPK 16‐4‐10) was added. The nitrogen level in the control treatment was assumed to be enough for optimal growth, that is, nitrogen was not a growth‐limiting nutrient in any of the treatments.

**TABLE 2:** Amount of nitrogen in pasteurized horse manure (PHM N, kg m^−3^) and additional fertilizer (added N, kg m^−3^) applied to the pots in different treatments at the beginning of the experiment and the total amount of nitrogen in the pots (N‐tot, kg m^−3^).

Treatment	PHM N (kg m^−3^)	Additional N (kg m^−3^)	N‐tot (kg m^−3^)
10% PHM	0.281	–	0.281
10% PHM + added N	0.281	0.039	0.320
20% PHM	0.563	–	0.563
30% PHM	0.844	–	0.844
40% PHM	1.125	–	1.125
Control treatment	–	0.320	0.320

All treatments were carried out with four replicates, with one treatment consisting of a total of 40 pots (10 pots per replicate). One seed of Chinese cabbage was sown in each pot. At the end of the experiment, the plants were cut from the surface of the growing media and weighed. Fresh weight was used to calculate the partial factor productivity of nitrogen (PFPN) using Equation (3). The method was modified from a method based on grain yield and described earlier by Cui et al. (2008). PFPN describes the use efficiency of fertilizer nitrogen for Chinese cabbage yield.

(3) $$\mathrm{PFPN}\;\left(g\;g^{-1}\right)=\frac{Yf}{NA}$$

where *Yf* is the yield of fresh biomass (g) at the end of the experiment and *NA* is the nitrogen fertilization (g) applied to the pots at the beginning of the experiment.

### 2.8 Statistical analysis

The effect of pasteurization on *E*. *coli* counts was analyzed using the Kruskal–Wallis Test, followed by non‐parametric pairwise comparisons using the Steel–Dwass Test. The effects of pasteurization, bedding material, and concentration of bedding material for AGR and ARLP in the phytotoxicity test were analyzed using multivariate analysis of variance (ANOVA). One‐way Anova was used for the statistical analysis of PFPN in the growing experiment. Tukey's test was used as a post hoc test for pairwise comparisons. The normal distribution and homogeneity of variances were tested using the Shapiro–Wilk and Levene tests using residuals. All the statistical analyses were performed using JMP Pro version 16.2.0 (SAS Institute Inc.) at a significance level of *p* < 0.05.

## 3 RESULTS AND DISCUSSION

### 3.1 Presence of *E. coli*

Pasteurization effectively reduced the bacterial counts in PHM with wood and peat bedding. The minimum and maximum counts of *E. coli* are presented in Table 3. The amount of *E. coli* after 24 h incubation in UHM was 5.2 × 10^4^–8.4 × 10^5^ CFU g^−1^, and significantly lower, <10–173 CFU g^−1^ in both PHMs (*p* < 0.05). Pasteurization successfully reduced the *E. coli* counts below the limit set by the Finnish Act on Fertilization Products (Fertilizer Product Act, 964/2023), where the maximum amount of *E. coli* is set at 10^3^ CFU g^−1^.

**TABLE 3:** *Escherichia coli* counts (colony‐forming unit [CFU] g^−1^) in untreated (UHM) and pasteurized horse manure (PHM) with either wood or peat bedding.

Treatment	Duration of incubation (h)	*N*	Minimum (CFU g^−1^)	Maximum
UHM with peat bedding	24	6a	5.2 × 10^4^	8.4 × 10^5^
PHM with peat bedding	24	6b	10	131
	48	6b	10	103
PHM with wood bedding	24	4b	<10	173
	48	4b	<10	198

*Note*: Batches in the same column not sharing the same letter are significantly different according to the Steel–Dwass test (*p* < 0.05) (*n* = 3; *N* = number of analyzed batches).

The presence of pathogens, including *E. coli*, is typical in animal slurries and manures. *Escherichia coli* is a human pathogen causing severe infections like diarrhea and hemorrhagic colitis (Pell, 1997), and thus its existence in growing media is strictly regulated by legislation. This study confirmed that pasteurization is an efficient method for reducing *E. coli* from horse manure. This is in accordance with previous studies showing pasteurization as an applicable method to reduce *E. coli* from animal slurry or manure (Michailidou et al., 2024; Watcharasukarn et al., 2009). Composting is a common method for reducing pathogens in the growing media industry. Compost composition and heap size effect on microbial activity (Shepherd et al., 2007). The reduction of *E*. *coli* through composting has ranged from 14 days (Berry et al., 2013) to 4 months (Shepherd et al., 2007). In comparison to composting, the clear advantage of pasteurization method described here is that it can eliminate pathogens rapidly, in only 1 h.

### 3.2 Seed viability after pasteurization

Pasteurization was effective in eliminating seed germination of cereals and weed seeds. No germination was observed in any of the pasteurized seeds (Table 4). Germination rates in the control treatments for the barley, oat, rye, wheat, or weed seed mixture were 92%, 93%, 97%, 97%, and 39%, respectively.

**TABLE 4:** Germination rate (%, mean ± SD) of 100 seeds of barley, oat, rye, wheat, or 1 g of weed seed mixture after pasteurization at >70°C for 60 min or without pasteurization (control) (*n* = 3).

Species	Pasteurization at >70°C (%)	Control
Barley	0 ± 0	92 ± 4
Oat	0 ± 0	93 ± 4
Rye	0 ± 0	97 ± 3
Wheat	0 ± 0^a^	97 ± 0
Weed seed mixture	0 ± 0	39 ± 7

^a^ One replicate did not reach the required time at >70°C (*n* = 2).

Concerns about the presence of diseases or weed seeds in reused growing media may hamper their release onto the market. This study showed that pasteurization is a promising method for reducing pathogens and seed viability existing in horse manure. Even shorter heat treatment time is efficient in elimination of seeds. For different weed seeds, heat treatment at 70°C for 10 or 40 min resulted in 100% mortality (Dahlquist et al., 2007). Similarly, seeds of *Brassica napus* L., *Solanum nigrum* L., and *Chenopodium album* L. incubated in pig slurry and heat‐treated at 75°C for 15 min eliminated seed viability to 0%, while heat treatment at 50°C for 15 min significantly reduced seed viability (Bloemhard et al., 1992).

### 3.3 Phytotoxicity of UHM or PHM

Pasteurization, bedding material, or the amounts of PHM or UHM in the growing media did not influence the AGRs in any of the treatments (*p* > 0.05). The AGRs in the treatments with 20% bedding materials (86%–96%) and in the control treatment (88%) were slightly higher, although not significantly, than those in the treatments containing 50% wood or peat bedding (88%–90%). The only exception was peat bedding with 20% UHM, where the AGR was slightly lower (86%) than in the treatment with 50% UMH (90%).

Generally, the low percentage (20%) of horse manure and the pasteurization had a positive effect on ARLP. There were also interaction effects between the bedding materials used, concentrations, and pasteurization (*p* < 0.05). The roots were longest (*p* < 0.05) in treatments containing 20% UHM with wood bedding (35 mm) and in the control treatment (33 mm), and comparable in treatments with 20% UHM or PMH with peat bedding. In the treatments containing 20% or 50% of the same material amendment, the higher amounts negatively affected (*p* < 0.05) ARLP in treatments containing PHM with peat bedding and UHM with wood bedding. In treatments containing 20% or 50% PHM with wood bedding and 50% UHM with wood bedding, the root lengths were statistically the same.

For the phytotoxicity test, the target value for MLV mentioned in the Finnish Act concerning Fertilization Products (Fertilizer Product Act, 964/2023) is set at 70%. Values below this are considered an indication of potential phytotoxic compounds in the tested material. This critical value was exceeded in all treatments with 20% UHM or PMH, except in the treatment containing PHM with wood bedding. The MLV was also above 70% in the treatment containing 50% PHM with wood bedding. Consequently, phytotoxic symptoms (MLV < 70%) were found in the treatments containing 50% UHM with wood bedding (66%), 50% UHM with peat bedding (62%), and 50% PHM with peat bedding (53%) (Table 5).

**TABLE 5:** Average germination rate (AGR %, mean ± SD), average root length per plant (ARLP mm, mean ± SD) and Munoo–Liisa vitality index (MLV %) in the peat control and treatments containing 20% or 50% untreated horse manure (UHM) or pasteurized horse manure (PHM) with wood or peat bedding.

Treatment	AGR (%)	ARPL (mm)	MLV (%)
Control treatment	88 ± 11a	33 ± 3a	101
20% UHM with wood bedding	94 ± 8a	35 ± 2a	112
50% UHM with wood bedding	82 ± 16a	24 ± 3cd	66
20% PHM with wood bedding	94 ± 5a	20 ± 3cd	62
50% PHM with wood bedding	90 ± 10a	25 ± 5bcd	76
20% UHM with peat bedding	86 ± 13a	27 ± 5abc	79
50% UHM with peat bedding	90 ± 12a	20 ± 1cd	62
20% PHM with peat bedding	96 ± 5a	32 ± 6ab	105
50% PHM with peat bedding	90 ± 10a	18 ± 5d	53

*Note*: Means in the same column not sharing the same letter are significantly different according to Tukey's test (*p* < 0.05) (*n* = 5).

The findings derived from ARLP and MLV indicate that 20% UHM or PHM with peat bedding is the tolerable amount for the growing media. A similar indication was found in a previous study where the 1:2 ratio of composted horse manure and peat was found to be too high for lettuce (*Lactuca sativa* L.) and cucumber (*Cucumis sativus* L.) (Vukobratović et al., 2018). When comparing bedding materials, 20% PHM with peat bedding was found to be considerably less phytotoxic than 20% PHM with wood bedding (MLV*s* 105 and 62%). Since phytotoxic symptoms were not obtained in 20% UHM with wood bedding, the release of phytotoxic compounds originated from wood material during pasteurization is probable. In Finland, wood bedding is a byproduct of the sawmill industry, primarily derived from pine (*Pinus sylvestris* L.) or spruce [*Picea abies* (L.) H. Karst.]. For example, essential oils containing terpenes and phenolic compounds, for example, ferulic acid and sinapic acid found in pine and spruce, have been shown to cause phytotoxic symptoms (Metsämuuronen & Sirén, 2019; Reigosa & Pazos‐Malvido, 2007; Ulukanli et al., 2014).

### 3.4 Fertilization effect of PHM with peat bedding

**FIGURE 3:**
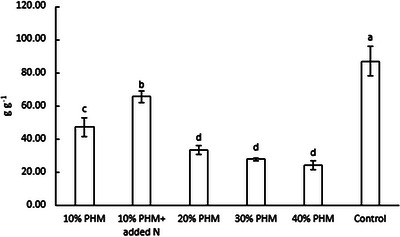
Partial factor productivity of nitrogen (PFPN g g^−1^, mean ± SD) of Chinese cabbage at the end of the experiment. PHM, pasteurized horse manure. Means not sharing the same letter are significantly different according to Tukey's test (*p* < 0.05) (*n* = 4).

During the experiment, not all of the nitrogen in the PHM was used for the growth of Chinese cabbage (Figure 3). The PFPN, which describes the efficiency of fertilizer nitrogen use, was lower in all treatments containing PHM than in the control treatment (87.15 g g^−1^) (*p* < 0.05). The additional nitrogen fertilizer in the 10% PHM treatment increased the PFPN (65.85 g g^−1^) compared to the other treatments containing PHM (*p* < 0.05).

**FIGURE 4:**
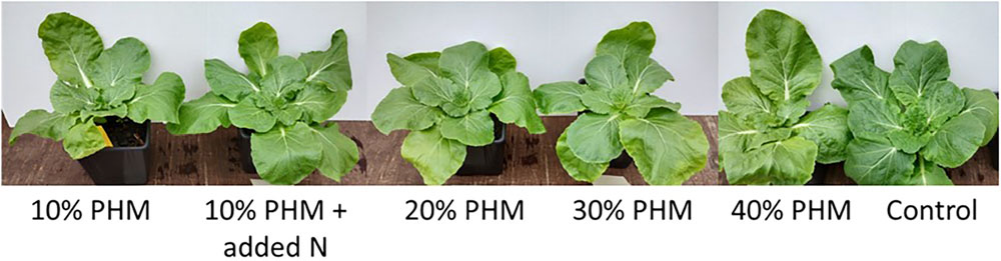
Chinese cabbages in treatments at the end of the experiment. PHM, pasteurized horse manure.

Nitrogen in horse manure is mostly in organic form, and nitrogen mineralization is essential for the use of horse manure as a fertilizer. The low PFPNs obtained in the PHM treatments, compared to the control treatment and the treatment with nitrogen supplementation, indicate that short‐term heat treatment did not efficiently mineralize the organic nitrogen, and only a minority of the nitrogen fraction was in a bioavailable form. However, the visual appearance of the plants (Figure 4) did not indicate nitrogen limitation in the treatments with 20%–40% PHM; that is, the plants were visually similar to those in the control treatment.

In the treatment with 10% PHM, the plant growth was weaker than in the other treatments, and nitrogen limitation was plausible. This suggests that regardless of low PFPNs in 20%–40% PHM treatments, the amount of bioavailable nitrogen was probably higher than in treatment containing less (10%) of PHM, and this was seen as better growth. Low nitrogen mineralization in combination with high salinity in composted horse manure with straw bedding has previously been reported and found to result in poor germination and yield (Clark & Cavigelli, 2005). However, composted horse manure with peat bedding has been successfully used as a long‐term fertilizer in greenhouse, and cow manure with wheat straw bedding as a long‐lasting supply of nitrogen in field conditions (Airaksinen, 2006; Pinto et al., 2017). Recycling PHM into a growing media has the potential to reduce the need for virgin materials and decrease the environmental impact of greenhouse cultivation. Thus, nitrogen mineralization and its release from horse manure in long‐term cultivation should be studied in the future to fully evaluate its suitability for greenhouse or field cultivation.

## 4 CONCLUSIONS

The sanitation of horse manure using a short‐term heat treatment was proved effective for eliminating *E. coli*, as well as cereal and weed seeds. Based on the results of phytotoxicity and growing experiments, PHM can be used to partially replace peat in growing media. Phytotoxic symptoms can occur if the amount of horse manure is high or if it has not been treated. After heat treatment, PHMs are very similar externally to the original bedding with no visible fecal balls, which is important in consumer use. The nitrogen absorbed in PHM was not used efficiently during short‐term cultivation, and additional nitrate‐nitrogen fertilization enhanced nitrogen uptake and growth. Further research should reveal the release of nitrogen from PMH in long‐term cultivation.

## AUTHOR CONTRIBUTIONS

**Salla Leppäkoski**: Conceptualization; data curation; formal analysis; investigation; methodology; visualization; writing—original draft. **Vilhelmiina Harju**: Methodology; writing—original draft. **Marika Tossavainen**: Data curation; formal analysis; writing—review and editing. **Ilpo Pölönen**: Conceptualization; funding acquisition; project administration; writing—review and editing.

## CONFLICT OF INTEREST STATEMENT

Ilpo Pölönen is the developer of the manure pasteurization device (ManPas) and a shareholder in ManPas Company, which is preparing to bring the device to market. This study involved testing an early prototype of ManPas. The authors declare this potential conflict of interest in the interest of transparency. The other three authors declare no conflicts of interest.
